# Trends in Hepatocellular Carcinoma Mortality Rates in the US and Projections Through 2040

**DOI:** 10.1001/jamanetworkopen.2024.45525

**Published:** 2024-11-18

**Authors:** Sikai Qiu, Jiangying Cai, Zhanpeng Yang, Xinyuan He, Zixuan Xing, Jian Zu, Enrui Xie, Linda Henry, Custis R. Chong, Esther M. John, Ramsey Cheung, Fanpu Ji, Mindie H. Nguyen

**Affiliations:** 1 Department of Infectious Disease, The Second Affiliated Hospital of Xi’an Jiaotong University, Xi’an, Shaanxi, China; 2 The Second Hospital and Clinical Medical School, Lanzhou University, Lanzhou, China; 3 School of Mathematics and Statistics, Xi’an Jiaotong University, Xi’an, Shaanxi, China; 4 Division of Gastroenterology and Hepatology, Department of Medicine, Stanford University Medical Center, Palo Alto, California; 5 Gastrointestinal Oncology Service, Department of Medicine, Memorial Sloan-Kettering Cancer Center, New York, New York; 6 Department of Epidemiology and Population Health, Stanford University School of Medicine, Stanford, California; 7 Division of Oncology, Department of Medicine, Stanford University School of Medicine, Stanford, California; 8 Stanford Cancer Institute, Stanford University School of Medicine, Stanford, California; 9 Division of Gastroenterology and Hepatology, Palo Alto Veterans Affairs Medical Center, Palo Alto, California; 10 Key Laboratory of Surgical Critical Care and Life Support, Xi’an Jiaotong University, Ministry of Education, Xi’an, Shaanxi, China; 11 Shaanxi Provincial Clinical Medical Research Center of Infectious Diseases, Xi’an, Shaanxi, China; 12 National and Local Joint Engineering Research Center of Biodiagnosis and Biotherapy, The Second Affiliated Hospital of Xi’an Jiaotong University, Xi’an, Shaanxi, China; 13 Key Laboratory of Environment and Genes Related to Diseases, Xi’an Jiaotong University, Ministry of Education, Xi’an, Shaanxi, China

## Abstract

**Importance:**

The burden of liver cancer varies worldwide. An upward trend in both hepatocellular carcinoma (HCC) incidence and mortality in the past 2 decades has been observed.

**Objective:**

To assess observed HCC-related age-standardized mortality rates (ASMRs) in the US for 2006 to 2022 and provide ASMR projections through 2040.

**Design, Setting, and Participants:**

This cross-sectional study used data from the National Vital Statistics System, which is accessible through the Centers for Disease Control and Prevention Wide-Ranging Online Data for Epidemiologic Research website. Data on deaths attributed to HCC (from January 1, 2006, to December 31, 2022) were obtained for adults 25 years or older and were stratified by liver disease etiology, age, sex, and race and ethnicity. Etiologies included alcohol-associated liver disease (ALD), hepatitis B virus (HBV), hepatitis C virus (HCV), and metabolic dysfunction–associated steatotic liver disease (MASLD).

**Main Outcomes and Measures:**

The main outcomes were (1) observed ASMRs of HCC per 100 000 persons using Joinpoint regression (National Cancer Institute) to assess trends during 2006 to 2022 and (2) ASMRs projected for 2023 to 2040 using Prophet and AutoARIMA modeling.

**Results:**

This study included 188 280 HCC-related deaths from 2006 to 2022. Most deaths occurred among males (77.4%). The annual percentage change was 4.1% (95% CI, 2.2% to 7.7%) for 2006 to 2009 and decreased to 1.8% (95% CI, 0.7% to 2.0%) for 2009 to 2022, with an overall observed ASMR of 5.03 per 100 000 persons in 2022 and a projected ASMR of 6.39 per 100 000 persons by 2040, with consistent trends for both sexes. By etiology, ASMRs decreased for HCV- and HBV-related mortality but increased for ALD- and MASLD-related mortality. In 2022, MASLD surpassed HBV as the third-leading cause of HCC-related death and was projected to overtake HCV in 2032 as the second-leading cause; ALD was projected to be the leading cause of HCC-related death in 2026. In 2022, the ASMR was higher among individuals aged 65 years or older compared with those aged 25 to 64 years (18.37 vs 1.79 per 100 000 persons). The American Indian or Alaska Native population had the largest increase in projected ASMR by 2040 (14.71 per 100 000 persons) compared with the Asian population (3.03 per 100 000 persons).

**Conclusions and Relevance:**

In this cross-sectional study, ASMRs for ALD- and MASLD-related HCC death increased rapidly from 2006 to 2022; ALD-related HCC was projected to be the leading cause by 2026, with MASLD as the second-leading cause by 2032. These findings may serve as a reference for public health decision-making and timely identification of groups at high risk of HCC death.

## Key Points

**Question:**

What is the trajectory of age-standardized mortality rates (ASMRs) associated with hepatocellular carcinoma (HCC), and what are the differences in HCC ASMRs by liver disease etiologies, sex, age, and race and ethnicity projected to 2040 in the US?

**Findings:**

In this cross-sectional study of 188 280 HCC-related deaths, ASMRs increased from 2006 to 2022 and were projected to continue rising until 2040, primarily due to increased deaths from alcohol-associated liver disease (ALD) and metabolic dysfunction–associated steatotic liver disease (MASLD); deaths from viral hepatitis were under control and were projected to decrease. Large disparities were observed in HCC-related ASMRs by age, sex, and race and ethnicity.

**Meaning:**

In this study, ALD and MASLD were projected to be the leading causes of HCC-related mortality by 2040 for most racial and ethnic groups; these findings may serve as a reference for public health decision-making and timely identification of groups at high risk of HCC-related death.

## Teaser

This cross-sectional study assesses trends in hepatocellular carcinoma (HCC)-related age-standardized mortality rates (ASMRs) by etiology, age, sex, and race and ethnicity in the US for 2006 to 2022 and provides ASMR projections to 2040.

## Introduction

Primary liver cancer imposes a substantial global disease burden, ranking as the sixth most commonly diagnosed cancer worldwide and the third-leading cause of global cancer-related mortality in 2020.^1^ Hepatocellular carcinoma (HCC) accounts for 75% to 85% of primary liver cancers and for the majority of liver cancer diagnoses and deaths.^2^ The similarity between HCC incidence and mortality underlines the poor prognosis associated with this disease.^2,3^

An upward trend in both HCC incidence and mortality has been observed over the past 2 decades.^4,5^ Although hepatitis B virus (HBV) and hepatitis C virus (HCV) infection remain pivotal risk factors for HCC incidence and mortality, advances in preventive HBV vaccines and antiviral therapies, including direct-acting antiviral therapy for HCV, have led to stabilization of and a decrease in HCC-related mortality rates from HBV and HCV infection. On the other hand, HCC cases related to alcohol-associated liver disease (ALD) and metabolic dysfunction–associated steatotic liver disease (MASLD) (formerly known as nonalcoholic fatty liver disease) are increasing.^2,5,6,7,8^ In the US, HCC mortality differs by age, sex, and race and ethnicity.^5,6,7^ The recent COVID-19 pandemic has also added challenges for the progress toward the World Health Organization goal of viral hepatitis elimination by 2030 through an unprecedented increase in alcohol consumption.^9,10,11,12^

Together, the aforementioned factors may alter the mortality trajectory associated with HCC over the next decades in US. Therefore, we examined trend data and future projections as well as differences in rates of HCC mortality rates by liver disease etiologies, sex, age, and race and ethnicity from 2006 to 2040.

## Methods

The data analyzed in this cross-sectional study were obtained from the National Vital Statistics System (NVSS) database, which is accessible through the Centers for Disease Control and Prevention (CDC) Wide-Ranging Online Data for Epidemiologic Research (WONDER) website.^13^ Institutional review board approval and the need for informed consent were waived in accordance with the Common Rule, because all data from the NVSS are fully deidentified. Detailed methods are presented in the eMethods in Supplement 1. The study followed the Strengthening the Reporting of Observational Studies in Epidemiology (STROBE) reporting guideline.

### Study Population and Design

We included data from the NVSS database on deaths attributed to HCC among adults 25 years or older in the US during from January 1, 2006, to December 31, 2022. We further stratified deaths based on liver disease etiology (ALD, HBV, HCV, or MASLD), age (25-64 or ≥65 years), sex (male or female), and race and ethnicity (self-reported as American Indian or Alaska Native, Asian or Native Hawaiian or Other Pacific Islander [hereinafter, Asian], Black, Hispanic, or White). Race and ethnicity was included because disparities have been observed in HCC-related mortality among racial and ethnic groups. However, these analyses were limited to the end of 2020 because the CDC revised race and ethnicity categories in 2021 to incorporate a new category for individuals identifying as multiple races, rendering the 2021 race and ethnicity data incompatible with data before 2021. The *International Statistical Classification of Diseases, Tenth Revision* (*ICD-10*) diagnostic codes for HCC and etiologies are listed in eTable 1 in Supplement 1.

### Statistical Analysis

We used Joinpoint Trend Analysis, version 5.0.2 (National Cancer Institute), to explore trends in age-standardized mortality rates (ASMRs) per 100 000 persons among individuals with HCC and to analyze ASMR annual percentage changes (APCs) with 95% CIs.^14^ The point that links 2 segments is defined as a joinpoint (eg, 2009 in temporal segment 2006-2009), determining whether the trend was better explained by 2 segments or more. To project HCC mortality rates, we used 2 time-series forecasting models (AutoARIMA and Prophet) to project ASMRs from 2023 to 2040 based on ASMRs in 2006 to 2022 (eTable 2 in Supplement 1). The threshold of significance was set at 2-sided *P* < .05. Statistical analysis was performed with R, version 4.0.2 (R Project for Statistical Computing).

## Results

### Population Characteristics

**Figure 1.**
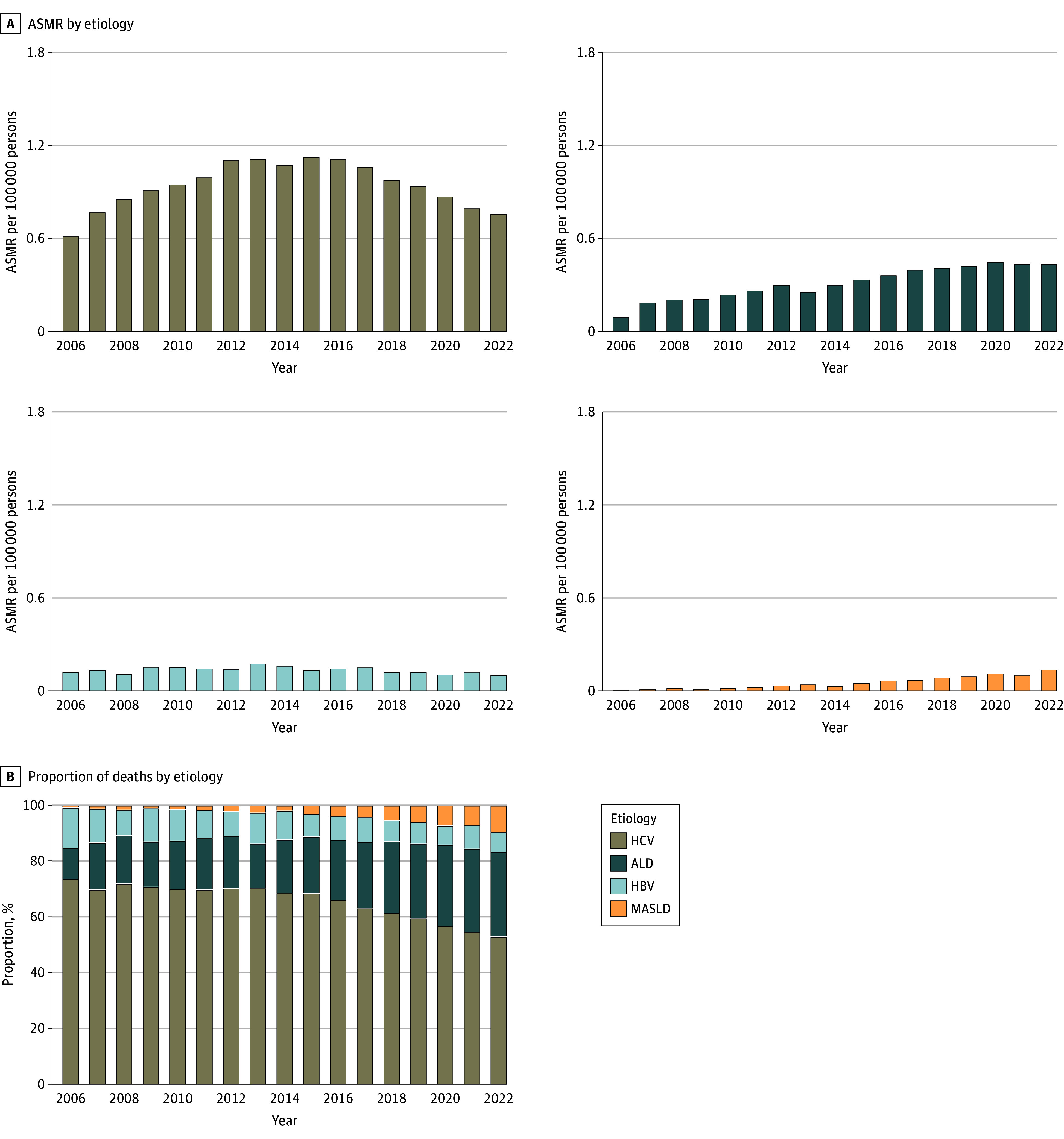
Cause of Death Related to Hepatocellular Carcinoma Presented by Age-Standardized Mortality Rate (ASMR) and Proportion by Different Etiologies ALD indicates alcohol-associated liver disease; HBV, hepatitis B virus; HCV, hepatitis C virus; MASLD, metabolic dysfunction–associated steatotic liver disease.

From 2006 to 2022, a total of 188 280 deaths among adults 25 years or older with HCC were documented (eTable 3 in Supplement 1). Most of these deaths (55.6%) occurred among older individuals (aged ≥65 years). The majority occurred among males (77.4%) compared with females (22.6%). In terms of race and ethnicity, 1.1% of deaths occurred among American Indian or Alaska Native individuals, 7.2% among Asian individuals, 15.5% among Black individuals, 13.9% among Hispanic individuals, and 62.3% among White individuals. As shown in Figure 1A, although the proportion of HBV-related HCC deaths decreased slowly between 2006 and 2022, the decrease in HCV-related HCC deaths occurred most rapidly after 2015. By contrast, the proportions of HCC deaths related to ALD and MASLD increased rapidly from 2006 to 2022. However, in 2022, HCV remained the most common etiology, accounting for 54.0% of deaths, followed by ALD (29.8%); MASLD (8.8%) surpassed HBV (7.4%) as the third-leading cause of death in 2022 (Figure 1B).

### Overall Trends and Projected HCC-Related ASMRs

**Figure 2.**
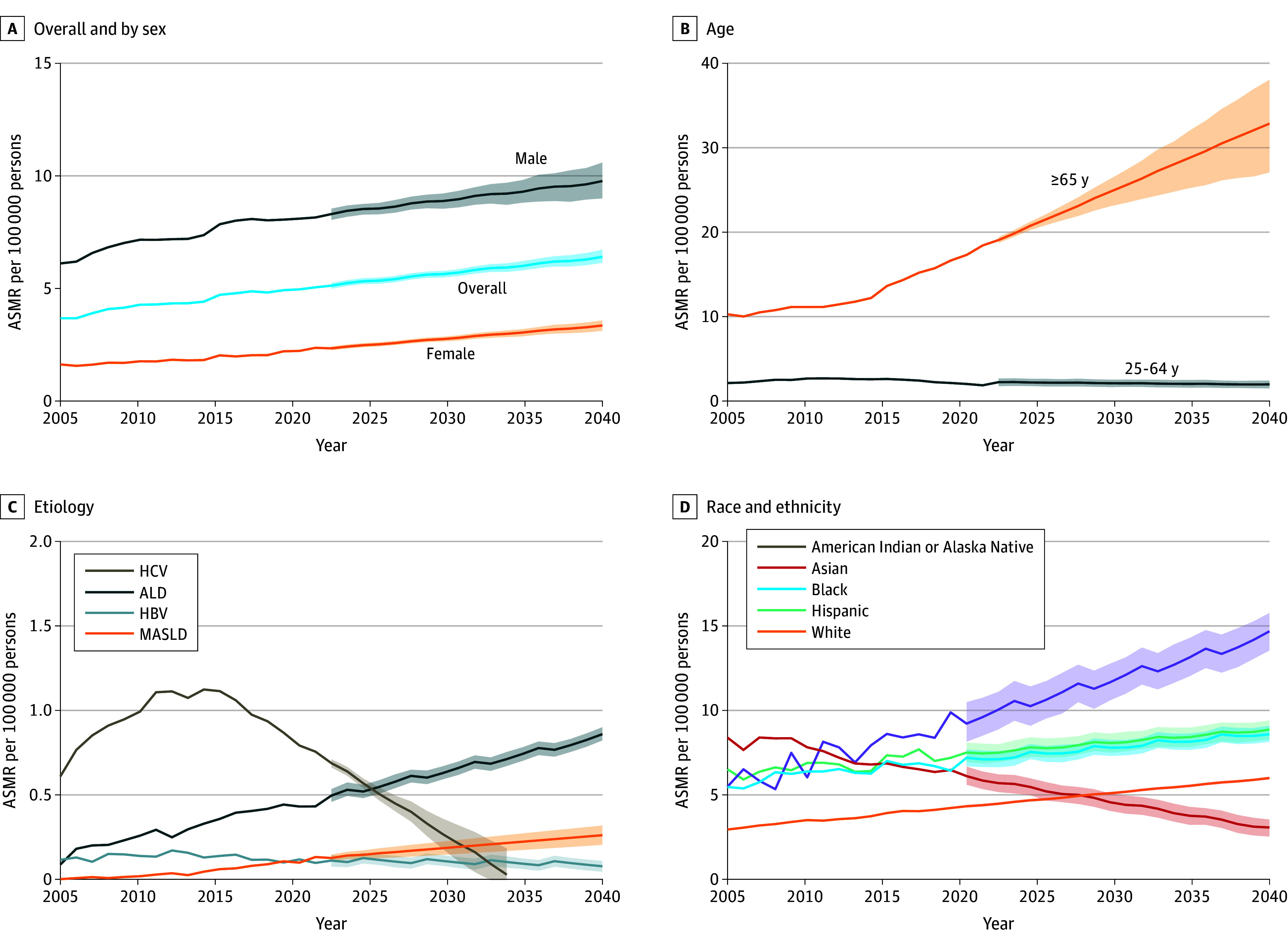
Observed and Projected Age-Standardized Mortality Rates (ASMRs) for Hepatocellular Carcinoma, Overall and by Sex, Age, Etiology, and Race and Ethnicity, 2006-2040 Shaded areas indicate 95% CIs. ALD indicates alcohol-associated liver disease; HBV, hepatitis B virus; HCV, hepatitis C virus; MASLD, metabolic dysfunction–associated steatotic liver disease.

Overall, the ASMR for HCC-related deaths increased from 3.65 per 100 000 persons in 2006 to 5.03 per 100 000 persons in 2022 (Table 1). The APC decreased from 4.1% (95% CI, 2.2% to 7.7%) for 2006 to 2009 to 1.8% (95% CI, 0.7% to 2.0%) for 2009 to 2022 on trend segment analysis. As shown in Figure 2A, we projected the overall ASMR for HCC-related deaths to increase to 6.39 per 100 000 persons by 2040 (eTable 4 in Supplement 1).

**Table 1.** APC and ASMR Among US Adults With Hepatocellular Carcinoma, Overall and By Sex, Age, and Etiology, 2006-2022

Characteristic	ASMR per 100 000 persons					Temporal trend difference		*P* value
	2006	2010	2015	2020	2022	Temporal segment^a^	APC (95% CI), %	
Overall	3.65	4.12	4.39	4.91	5.03	2006-2009	4.1 (2.2 to 7.7)	<.001
						2009-2022	1.8 (0.7 to 2.0)	.04
Sex								
Female	1.59	1.66	1.79	2.18	2.33	2006-2019	2.2 (0 to 4.0)	.05
						2019-2022	4.7 (2.3 to 8.1)	<.001
Male	6.10	7.01	7.37	8.06	8.15	2006-2010	4.0 (3.3 to 4.9)	<.001
						2010-2014	0.4 (−0.5 to 1.1)	.33
						2014-2017	3.7 (2.6 to 4.5)	<.001
						2017-2022	0.2 (−0.4 to 0.6)	.42
Age, y								
25-64	2.06	2.43	2.51	2.07	1.79	2006-2011	4.9 (3.9 to 6.1)	<.001
						2011-2017	−0.8 (−1.7 to 0)	.06
						2017-2022	−6.2 (−7.2 to −5.2)	<.001
≥65	10.20	11.08	12.15	16.59	18.37	2006-2014	1.8 (0.9 to 2.8)	.01
						2014-2017	7.2 (1.0 to 8.3)	.01
						2017-2022	5.0 (3.0 to 5.8)	<.001
Etiology								
HCV	0.61	0.95	1.12	0.87	0.76	2006-2008	17.2 (11.9 to 23.2)	<.001
						2008-2012	5.9 (2.5 to 7.6)	.004
						2012-2016	1.0 (−8.8 to 2.6)	.53
						2016-2022	−6.4 (−8.5 to −3.4)	.002
ALD	0.09	0.23	0.33	0.44	0.43	2006-2008	45.1 (19.9 to 72.6)	<.001
						2008-2022	5.9 (4.0 to 7.2)	.01
HBV	0.12	0.15	0.13	0.10	0.10	2006-2013	4.4 (0.7 to 16.5)	.02
						2013-2022	−4.8 (−10.4 to −2.3)	<.001
MASLD	0.01	0.02	0.05	0.11	0.14	2006-2022	19.0 (15.8 to 22.4)	<.001

Abbreviations: ALD, alcohol-associated liver disease; APC, annual percentage change; ASMR, age-standardized mortality rate; HBV, hepatitis B virus; HCV, hepatitis C virus; MASLD, metabolic dysfunction–associated steatotic liver disease.

^a^ The point that links 2 segments is defined as a joinpoint, determining whether the trend was better explained by 2 segments or more.

#### Sex

Increasing trends in mortality from 2006 to 2022 were similar for both males and females; however, the ASMR for males was higher than that of females (8.15 vs 2.33 per 100 000 persons in 2022). The ASMR increased steadily for females, with the APC increasing from 2.2% (95% CI, 0% to 4.0%) in 2006 to 2019 to 4.7% (95% CI, 2.3% to 8.1%) in 2019 to 2022. The ASMR was projected to increase to 3.33 per 100 000 persons by 2040 (Figure 2A). By contrast, the growth rate slowed after 2017 for males, with a projected ASMR of 9.78 per 100 000 persons by 2040 (Figure 2A).

#### Age

For the group aged 25 to 64 years, the ASMR for HCC-related deaths increased from 2006 to 2011 (APC, 4.9% [95% CI, 3.9%-6.1%]), followed by a small decrease from 2011 to 2017 (APC, −0.8% [95% CI, −1.7% to 0%]) and then a statistically significant decrease from 2017 to 2022 (APC, −6.2% [95% CI, −7.2% to −5.2%]; *P* < .001). The ASMR in this age group was projected to stabilize from 2023 to 2040 (Figure 2B).

For the group aged 65 years or older, the ASMR increased from 2006 to 2022, especially after 2014, yielding APCs of 7.2% (95% CI, 1.0%-8.3%) in 2014 to 2017 and 5.0% (95% CI, 3.0%-5.8%) in 2017 to 2022 (Table 1). The ASMR in the older age group was projected to increase from 18.37 per 100 000 persons in 2022 to 32.81 per 100 000 persons by 2040. In 2022, the ASMR of the group aged 65 years or older was 10 times higher than that of the group aged 25 to 64 years (18.37 vs 1.79 per 100 000 persons), a trend that was projected to continue to increase from 2023 to 2040 (Figure 2B).

#### Etiology

For HCV, the ASMR increased significantly from 2006 to 2016, with the fastest increase in 2006 to 2008 (APC, 17.2% [95% CI, 11.9% to 23.2%]; *P* < .001), followed by smaller increases in 2008 to 2012 (APC, 5.9% [95% CI, 2.5% to 7.6%]) and in 2012 to 2016 (APC, 1.0% [95% CI, −8.8% to 2.6%]). From 2016 to 2022, the ASMR decreased at a rate of 6.4% (95% CI, −8.5% to −3.4%) and was projected to decline from 0.69 per 100 000 persons in 2023 to 0.03 per 100 000 persons in 2034. For HBV, we also observed an overall upward and then a downward trend across the study period. However, the ASMR for HBV was projected to be more stable from 2023 to 2040 (Figure 2C), with a projected decrease to 0.08 per 100 000 persons by 2040.

By contrast, both ALD- and MASLD-related HCC had a general upward trend in ASMR from 2006 to 2022, with continued annual increases projected from 2023 to 2040. Over the period from 2006 to 2022, the average annual growth rate for ALD slowed, with the APC decreasing from 45.1% (95% CI, 19.9% to 72.6%) in 2006 to 2008 to 5.9% (95% CI, 4.0% to 7.2%) in 2008 to 2022. For MASLD, there was a steady increase in the ASMR, with an annual increase of 19.0% and no statistically significant segmental changes. It was projected that by 2040, the ASMR will increase to 0.86 per 100 000 persons for ALD and 0.26 per 100 000 persons for MASLD. Notably, ALD, MASLD, and HBV were also projected to overtake HCV as the first, second, and third causes of death from HCC starting in 2026, 2032, and 2033, respectively (Figure 2C).

#### Race and Ethnicity

Overall observed and projected HCC-related ASMRs increased from 2006 to 2020 and from 2023 to 2040, respectively, among American Indian or Alaska Native, Black, Hispanic, and White populations (Figure 2D). Of these, the largest increase was observed for the American Indian or Alaska Native population, with the ASMR projected to increase to 14.71 per 100 000 persons by 2040. By contrast, a decrease was observed for the Asian population, with the ASMR projected to decrease to 3.03 per 100 000 persons by 2040 (eTable 3 in Supplement 1).

### Subgroup Analysis and Projected HCC-Related ASMRs by Etiology

#### Etiology and Sex

For ALD-related HCC from 2006 to 2022, there was an overall increase in ASMR with a stable trend in 2008 to 2022 for both males (APC, 5.9% [95% CI, 3.2% to 7.5%]) and females (APC, 6.7% [95% CI, −5.5% to 10.7%]), following a larger initial increase (Table 2). The ASMR was projected to increase to 1.63 per 100 000 persons for males and 0.18 per 100 000 persons for females by 2040. Similarly, for MASLD-related HCC, we observed a sharp increase in ASMR for both males and females, with APCs of 19.2% (95% CI, 17.2% to 21.3%) and 17.8% (95% CI, 14.4% to 21.1%), respectively. The ASMR was projected to increase to 0.32 per 100 000 persons for males and to 0.21 per 100 000 persons for females by 2040.

**Table 2.** APC and ASMR Among US Adults With Hepatocellular Carcinoma, Stratified by Sex, Etiology, and Age, 2006-2022

Characteristic	ASMR per 100 000 persons (No. of deaths)					Temporal trend difference		*P* value
	2006	2010	2015	2020	2022	Temporal segment^a^	APC (95% CI), %	
**Sex**								
Female								
HCV	0.23 (241)	0.33 (395)	0.36 (521)	0.32 (505)	0.28 (43)	2006-2013	8.2 (6.2 to 10.8)	<.001
						2013-2022	−3.8 (−5.2 to −2.5)	<.001
ALD	0.01 (26)	0.04 (53)	0.06 (87)	0.08 (138)	0.09 (145)	2006-2008	92.2 (14.2 to 157.7)	<.001
						2008-2022	6.7 (−5.5 to 10.7)	.12
HBV	0.02 (34)	0.03 (53)	0.04 (47)	0.04 (56)	0.04 (58)	2006-2022	1.9 (−1.6 to 5.5)	.29
MASLD	Suppressed	0.02 (18)	0.04 (60)	0.09 (145)	0.11 (166)	2007-2022	17.8 (14.4 to 21.1)	<.001
Male								
HCV	1.00 (1028)	1.59 (1816)	1.96 (2531)	1.47 (2110)	1.28 (1888)	2006-2008	20.4 (12.8 to 28.5)	<.001
						2008-2015	4.7 (2.8 to 5.9)	.003
						2015-2022	−6.2 (−7.7 to −4.9)	<.001
ALD	0.16 (175)	0.46 (525)	0.62 (789)	0.83 (1132)	0.82 (1136)	2006-2008	49.4 (17.2 to 84.7)	<.001
						2008-2022	5.9 (3.2 to 7.5)	.02
HBV	0.20 (194)	0.27 (285)	0.25 (282)	0.22 (259)	0.21 (259)	2006-2010	5.0 (−0.2 to 23.6)	.07
						2010-2022	−2.0 (−8.8 to −0.9)	.02
MASLD	0.01 (10)	0.02 (25)	0.06 (73)	0.15 (213)	0.16 (213)	2006-2022	19.2 (17.2 to 21.3)	<.001
**Age, y**								
25-64								
HCV	0.55 (958)	0.88 (1728)	0.91 (2084)	0.51 (1238)	0.37 (914)	2006-2008	18.2 (11.6 to 25.4)	<.001
						2008-2012	5.4 (0.1 to 7.6)	.05
						2012-2016	−1.7 (−16.1 to 0.2)	.06
						2016-2022	−13.8 (−15.7 to −11.0)	<.001
ALD	0.06 (116)	0.23 (457)	0.26 (584)	0.28 (648)	0.26 (619)	2006-2008	70.6 (27.2 to 123.3)	<.001
						2008-2022	3.1 (0.4 to 5.0)	.04
HBV	0.09 (148)	0.12 (223)	0.10 (207)	0.08 (159)	0.08 (167)	2006-2013	5.6 (0.2 to 29.6)	.04
						2013-2022	−6.4 (−17.0 to −3.0)	.002
MASLD	Suppressed	0.01 (15)	0.02 (37)	0.03 (73)	0.03 (73)	2010-2022	14.3 (9.1 to 19.9)	<.001
≥65								
HCV	0.86 (311)	1.22 (483)	1.98 (968)	2.32 (1377)	2.34 (1405)	2006-2016	8.6 (7.3 to 11.6)	<.001
						2016-2022	2.4 (−3.7 to 5.1)	.17
ALD	0.23 (85)	0.30 (121)	0.62 (292)	1.06 (622)	1.14 (662)	2006-2022	10.6 (9.4 to 11.8)	<.001
HBV	0.22 (80)	0.29 (115)	0.25 (122)	0.28 (156)	0.28 (150)	2006-2022	0.8 (−0.3 to 2.1)	.16
MASLD	Suppressed	0.07 (28)	0.19 (96)	0.50 (285)	0.56 (306)	2007-2022	20.0 (17.7 to 22.3)	<.001

Abbreviations: ALD, alcohol-associated liver disease; APC, annual percentage change; ASMR, age-standardized mortality rate; HBV, hepatitis B virus; HCV, hepatitis C virus; MASLD, metabolic dysfunction–associated steatotic liver disease.

^a^ The point that links 2 segments is defined as a joinpoint, determining whether the trend was better explained by 2 segments or more.

**Figure 3.**
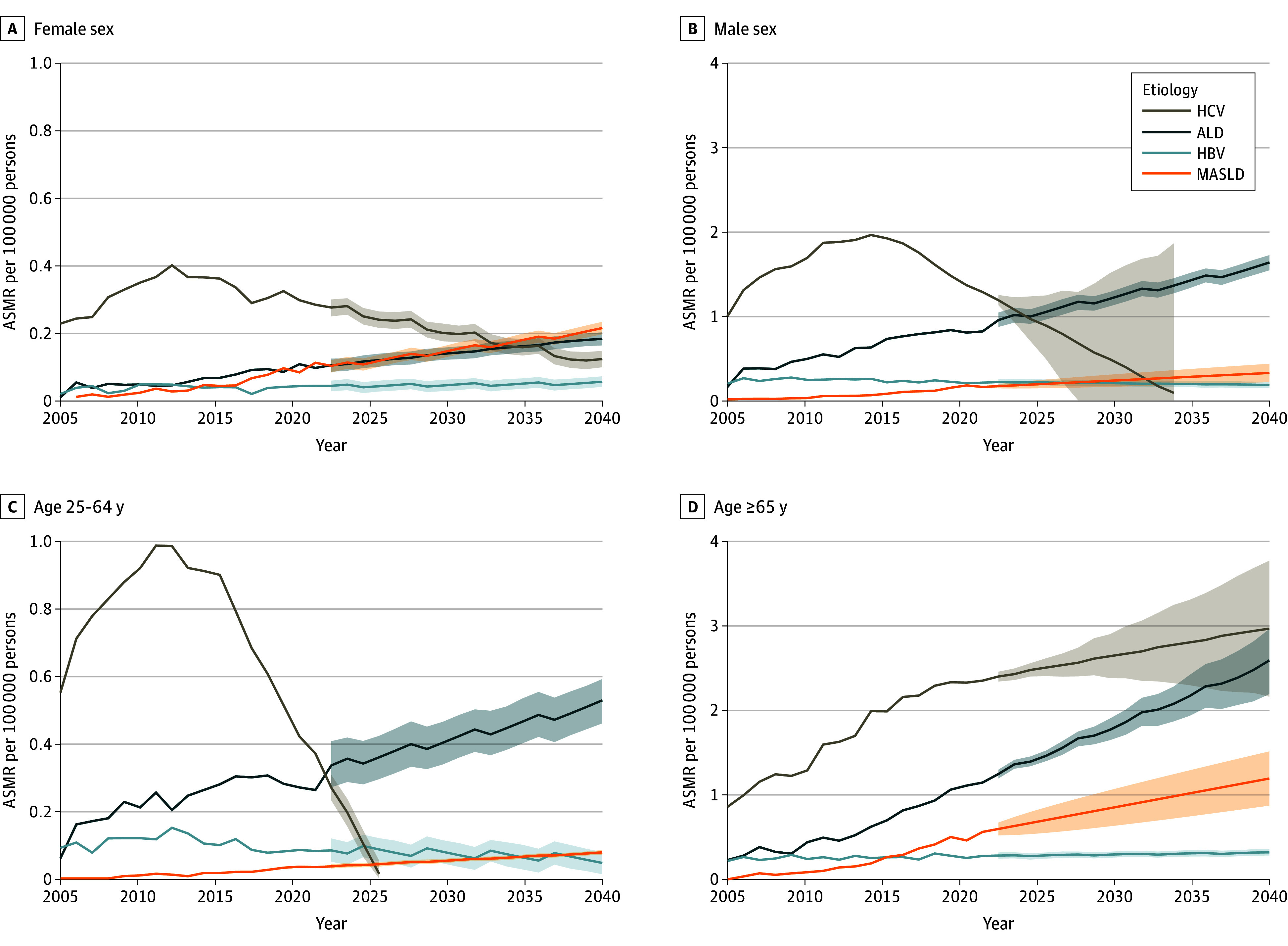
Observed and Projected Age-Standardized Mortality Rates (ASMRs) for Hepatocellular Carcinoma, by Sex, Age, and Etiology, 2006-2040 Projections for hepatitis C virus (HCV) to 2040 are based on data from 2015 to 2020. Projections for the other groups to 2040 are based on data from 2006 to 2022. Shaded areas indicate 95% CIs. ALD indicates alcohol-associated liver disease; HBV, hepatitis B virus; MASLD, metabolic dysfunction–associated steatotic liver disease.

By contrast, for HCV-related HCC for both sexes, we initially observed upward trends in ASMR, followed by downward trends. In particular, the ASMR for females was projected to decrease from 0.27 per 100 000 persons in 2023 to 0.12 per 100 000 persons by 2040 (Figure 3A); for males, it was projected to decrease from 1.18 per 100 000 persons in 2023 to 0.09 per 100 000 persons in 2034 (Figure 3B).

For HBV, after a nonsignificant increase for males from 2006 to 2010, the ASMR decreased significantly after 2010 (APC, −2.0% [95% CI, −8.8% to −0.9%]; *P* = .02) and was projected to decrease to 0.18 per 100 000 persons by 2040 (Table 1 and eTable 4 in Supplement 1). The ASMR for females was stable across the entire study period, with a projected ASMR of 0.05 per 100 000 persons by 2040. Notably, ALD was projected to overtake HCV as the leading cause of HCC-related deaths among males by 2026, whereas MASLD was projected to be the leading cause among females by 2034.

#### Etiology and Age

ASMRs for ALD in both age groups (25-64 and ≥65 years) increased significantly from 2006 to 2022 and were projected to increase further to 0.53 per 100 000 persons and 2.58 per 100 000 persons by 2040, respectively (25-64 years, *P* < .001 [2006-2008] and *P* = .04 [2008-2022]; ≥65 years, *P* < .001 [2006-2022]). Additionally, ALD was projected to overtake HCV as the leading cause of HCC-related deaths among persons aged 25 to 64 years, with an ASMR increase of 0.34 vs 0.27 per 100 000 persons in 2023.

For MASLD, there was a similar upward trend in ASMRs for both age groups (25-64 and ≥65 years), which were projected to increase to 0.08 per 100 000 persons and to 1.19 per 100 000 persons by 2040, respectively. The APCs were 14.3% (95% CI, 9.1% to 19.9%) for those aged 25 to 64 years and 20.0% (95% CI, 17.7% to 22.3%) for those aged 65 years or older, with no statistically significant segmental changes.

For HCV, there was an increasing and then decreasing trend in the ASMR among the group aged 25 to 64 years from 2006 to 2022, which was projected to decrease to 0.01 per 100 000 persons in 2026. However, the ASMR for the group aged 65 years or older increased across the study period and was projected to increase to 2.95 per 100 000 persons by 2040.

For HBV, there was also an increasing and then a decreasing trend among the group aged 25 to 64 years from 2006 to 2022, whereas no statistically significant change was observed for the group aged 65 years or older. For the group aged 25 to 64 years, the ASMR was projected to continue to decrease to 0.05 per 100 000 persons by 2040. By contrast, a small increase was projected for the older age group, with a projected ASMR of 0.32 per 100 000 persons by 2040 (Figure 3C and D and eTable 4 in Supplement 1).

#### Etiology and Race and Ethnicity

From 2006 to 2022, we observed an increase in the MASLD-related ASMR among Hispanic individuals and White individuals (eFigure, A and B in Supplement 1). The projected ASMR is 0.44 per 100 000 persons for Hispanic individuals and 0.28 per 100 000 persons White individuals by 2040, respectively.

For HCV, after an initial increase in ASMR, a consistent decrease was observed for all racial and ethnic groups. ASMRs for the Asian, Black, and Hispanic populations were projected to fall to very low levels after 2029, 2031, and 2034, respectively (eFigure, B-D in Supplement 1).

For HBV, the ASMR for the Asian population initially increased and peaked in 2010 (1.91 per 100 000 persons), then decreased afterward (eFigure, D in Supplement 1). However, it was projected to remain the leading cause of HCC-related death among Asian individuals through 2040. There were no statistically significant changes in the ASMRs for HBV among Black individuals or White individuals.

For ALD, the ASMR increased to varying degrees for all racial and ethnic groups, with the largest projected increase observed among American Indian or Alaka Native individuals, overtaking HCV as the leading cause of HCC-related deaths and reaching a projected 3.47 per 100 000 persons by 2040 (eFigure, E in Supplement 1). ALD was also projected to surpass HCV as the leading cause of HCC-related deaths for the Black and Hispanic populations in 2030 and for the White population in 2034.

## Discussion

In this study, we assessed trends in HCC mortality rates in the US from 2006 to 2022, from which we projected ASMRs for 2023 to 2040. We observed that the HCC mortality rate increased from 2006 to 2022. In regard to trends by etiology, the HCV-related HCC mortality rate has been decreasing since its peak in 2015, whereas the opposite trend was observed with ALD- and MASLD-related HCC mortality. Our findings suggest that ALD will surpass HCV to become the leading etiology of HCC-related deaths by 2026, whereas MASLD surpassed HBV to become the third-leading cause of HCC-related deaths in 2022 and will surpass HCV to become the second-leading cause in 2032. However, HBV was projected to remain the leading cause of HCC-related mortality among Asian individuals. We also found several other important disparities by sex, age, and race and ethnicity, with significant upward trends in HCC-related mortality among females, among the population aged 65 years or older (as opposed to a decreasing trend for the younger population), and among the American Indian and Alaska Native population, which had the largest upward trend in mortality.

The downward trend we observed in viral HCC-related mortality rates is likely due to the availability of effective and well-tolerated oral antiviral therapy since 2005 and 2015 for HBV and HCV, respectively, although less effective oral agents had been available for HBV since 1999. Antiviral therapy has been shown to not only reduce the incidence of HCC among patients with chronic HBV or HBC but also to substantially improve overall survival, recurrence-free survival, and liver-related survival among those with HBV- or HCV-related HCC.^15,16,17,18,19,20,21,22,23,24^ For HBV, an effective preventive vaccine has been in place for several decades, which has contributed to the success of primary preventive programs for HBV and would be consistent with the trend observed in our study of decreasing HBV-related HCC mortality rates for people younger than 65 years but not older adults.^6^ Similarly, we also found a decreasing trend in HCV-related HCC mortality among the group aged 25 to 64 years, whereas we observed an increased trend for those aged 65 years or older. This finding is likely due to the fact that effective oral antiviral therapy for HCV only became available toward the end of 2014, so many older patients with HCV infection probably also had established cirrhosis, which would place these patients at higher risk of developing HCC and at higher risk of death once they develop HCC due to poor hepatic function with cirrhosis. Also, in previous studies, HCC risk was reduced but persisted among patients with HCV and advanced fibrosis even after cure.^25,26^ Additionally, older people have a higher risk for metabolic disease such as diabetes and MASLD, both of which can independently increase the risk of HCC development and are very prevalent.^27,28,29,30^ Thus, it is important to increase screening for HBV and HCV, to initiate antiviral treatment for all patients who meet recommended treatment criteria, and to offer antiviral therapy for patients with HCV- or HBV-related HCC who do not have limited life expectancy that cannot be remediated by antiviral treatment.

It should be noted, however, that HBV remains the leading cause of HCC-related death among Asian individuals despite a decreasing rate overall. HCC-related mortality rates are still rising for females with HBV, opposite of the declining trend seen in males. The reasons for these observations, despite availability of an effective preventive vaccine for several decades as well as several effective and well-tolerated oral antiviral therapeutic options for over 2 decades, lie with the poor care cascade for people with chronic HBV infection. Previous studies have found that only approximately 18% of US patients with HBV infection have been diagnosed, only 30% to 60% of diagnosed patients had undergone a basic evaluation for treatment eligibility, and only 50% to 80% of treatment-eligible patients initiated antiviral treatment, with lower rates among females, Asian individuals, community practice, and nongastroenterology and noninfectious disease subspecialty practices.^19,31,32,33^ Thus, additional community, patient, and physician education and outreach is needed to optimize treatment of patients with chronic HBV infection, with the aim of reducing HCC incidence and HCC-related deaths.

Even more efforts are needed to curtail the alarming increase in mortality associated with ALD-related HCC, which we projected to become the number one cause of HCC-related deaths in the US in 2026. This trend also affects females and people older than 65 years. Alcohol consumption in the US has increased in recent years, especially during the COVID-19 pandemic, with females and older adults found to be more prone to binge drinking than men and younger adults.^34,35^ Females are also more likely to develop ALD with a lower level of alcohol consumption because they have lower gastric oxidation of ethanol, resulting in higher bioavailability of ethanol.^36^ ALD-related HCC mortality has also disproportionately affected the American Indian or Alaska Native population. In addition to the high prevalence of alcohol use disorder among American Indian or Alaska Native individuals, the high ALD-related HCC mortality rate seen in this population in this study may also be related to poor access to health care services, because many live in rural remote areas, and there are challenges with the low penetration of alcohol restriction policies in tribal autonomous and remote areas.^37,38,39^ Prior studies have reported a lower rate of HCC surveillance in patients with ALD compared with patients with cirrhosis from other liver disease etiologies, which likely contributes to the higher mortality associated with ALD-related HCC overall.^40,41^

Adherence to HCC surveillance is also poorer for patients with MASLD-related cirrhosis,^41^ which can lead to advanced-stage cancer and poorer survival in patients with MASLD-related HCC. Therefore, to curtail the rising mortality from MASLD-related HCC, which we projected to become the second-leading cause of HCC death in 2032 in the current study, additional efforts are also needed to improve HCC surveillance rates among people with MASLD. Moreover, prevention of MASLD development and MASLD disease progression should be a top public health priority, because one-third of the world population had MASLD as of 2019, and this rate was projected to increase to more than 50% by 2040.^27,42,43^

### Strengths and Limitations

The strength of this study is its use of the NVSS database as curated and maintained by the CDC, which includes data on more than 99% of deaths in the US. This data source allowed us to accurately calculate trends in HCC-related mortality rates in 2006 to 2022 and to make accurate projections for the future up to 2040, especially considering conflicting results by earlier reports based on only a sample of the population.^44,45^

This study has several limitations. First, the NVSS database only records patient deaths without individual medical records and detailed clinical data. Second, due to the inability to obtain anonymous individual data, our study did not analyze socioeconomic status or other individual-level risk factors. Third, the *ICD-10* code for nonalcoholic fatty liver disease was used to define MASLD in our investigation, because there are not yet any *ICD* codes for MASLD. However, prior studies have shown that there is much concordance between the 2 terms.^46^ Fourth, the study period also included the COVID-19 pandemic, which could have affected the trends observed and made future projections less reliable. Development of HBV treatment with a functional cure would also affect trends, as linkage to care may improve with a finite course of treatment and a functional cure is associated with lower risk of HCC.^47^ The 2024 approval of resmetirom, the first medication for the treatment of MASLD with stage 2 to 3 fibrosis, and additional future agents may help lower the incidence of MASLD-associated HCC and HCC-related deaths.^48^ Thus, therapeutic advances for MASLD and potential acceleration in the obesity epidemic both can affect our projections.^49,50^ The effectiveness and advances of an HCC surveillance program may also be enhanced by future availability of better HCC surveillance testing based on cell-free DNA tumor markers, and these advances would help improve survival of patients with HCC.^51^ Additionally, the straight-line segments assumed by Joinpoint regression are an approximation and cannot be distinguished from gradual curve trajectories over time.

## Conclusions

In this cross-sectional study, HCC mortality was projected to continue increasing in the US, primarily due to rising rates of deaths attributable to ALD and MASLD. Large disparities were observed in HCC-related mortality by age, sex, and race and ethnicity. This information offers valuable insights to help improve HCC prevention and treatment efforts, and these findings may serve as a reference for public health decision-making and timely identification of groups at high risk of HCC death.

## Notes

Supplement 1.
eMethods

eReferences
**eTable 1.** *International Statistical Classification of Diseases, Tenth Revision* (*ICD-10*), Codes Used in the Study **eTable 2.** Root Mean Square Error (RMSE) Comparison and Model Selection Between AutoARIMA and Prophet Across Different Groups **eTable 3.** Characteristics of Hepatocellular Carcinoma (HCC)–Related Deaths in the US, 2006-2022 **eTable 4.** Observed and Projected Age-Standardized Mortality Rates (per 100 000) of Adults (Aged ≥25 years) With Hepatocellular Carcinoma (HCC), US, 2006-2040, Total and by Sex, Age, and Race and Ethnicity, and Further Stratified by HCC Etiology **eFigure.** Causes of Deaths Related to Hepatocellular Carcinoma (HCC) Presented by the Age-Standardized Mortality Rate (ASMR) per 100 000 and Predicted Value to 2040, Overall and by Race and Ethnicity

Supplement 2.
Data Sharing Statement
