# Supplementary material for: Trends in Hepatocellular Carcinoma Mortality Rates in the US and Projections Through 2040
Source: JAMA Netw Open. 2024 Nov 18;7(11):e2445525. doi: 10.1001/jamanetworkopen.2024.45525 (PMC11574689; doi:10.1001/jamanetworkopen.2024.45525)
Supplement: Supplement 1. — eMethods eReferences eTable 1. International Statistical Classification of Diseases, Tenth Revision (ICD-10), Codes Used in the Study eTable 2. Root Mean Square Error (RMSE) Comparison and Model Selection Between AutoARIMA and Prophet Across Different Groups eTable 3. Characteristics of Hepatocellular Carcinoma (HCC)–Related Deaths in the US, 2006-2022 eTable 4. Observed and Projected Age-Standardized Mortality Rates (per 100 000) of Adults (Aged ≥25 years) With Hepatocellular Carcinoma (HCC), US, 2006-2040, Total and by Sex, Age, and Race and Ethnicity, and Further Stratified by HCC Etiology eFigure. Causes of Deaths Related to Hepatocellular Carcinoma (HCC) Presented by the Age-Standardized Mortality Rate (ASMR) per 100 000 and Predicted Value to 2040, Overall and by Race and Ethnicity [file jamanetwopen-e2445525-s001.pdf]

Supplementary Online Content

Qiu S, Cai J, Yang Z, et al. Trends in hepatocellular carcinoma mortality rates in the US and projections through 2040. *JAMA Netw Open*. 2024;7(11):e2445525. doi:10.1001/jamanetworkopen.2024.45525

eMethods

eReferences

eTable 1. *International Statistical Classification of Diseases, Tenth Revision* (*ICD-10*), Codes Used in the Study

eTable 2. Root Mean Square Error (RMSE) Comparison and Model Selection Between AutoARIMA and Prophet Across Different Groups

eTable 3. Characteristics of Hepatocellular Carcinoma (HCC)–Related Deaths in the US, 2006-2022

eTable 4. Observed and Projected Age-Standardized Mortality Rates (per 100 000) of Adults (Aged ≥25 years) With Hepatocellular Carcinoma (HCC), US, 2006-2040, Total and by Sex, Age, and Race and Ethnicity, and Further Stratified by HCC Etiology

eFigure. Causes of Deaths Related to Hepatocellular Carcinoma (HCC) Presented by the Age-Standardized Mortality Rate (ASMR) per 100 000 and Predicted Value to 2040, Overall and by Race and Ethnicity

This supplementary material has been provided by the authors to give readers additional information about their work.

© 2024 Qiu S et al. *JAMA Network Open.*

eMethods

The data were obtained from the National Vital Statistics System (NVSS) database. This comprehensive database encompasses more than 99% of deaths of United States residents across the 50 states and the District of Columbia. The database is continually updated and includes records through December 31, 2023. Each entry in the database corresponds to the mortality data of one individual. Pertinent demographic information such as age, sex, race and ethnicity, and etiology of death was extracted for analysis. All causes of death were documented using the 10th edition of the International Classification of Diseases. For the purposes of this study, the etiologies of HCC (C22.0) included HCV infection (B17.1, B18.2), HBV infection (B16, B17.0, B18.0, B18.1), ALD (K70), and MASLD (K75.8, K76.0).

We present the demographic characteristics of deceased individuals with HCC in terms of frequencies and percentages. We utilized the age structure (25 to ≥85 years) from the 2000 United States Census Standard Population and employed direct standardization to calculate age-standardized mortality rates (ASMRs per 100,000 persons).

We analyzed overall deaths from HCC and further stratified them by subgroup based on etiology (HCV, HBV, ALD, and MAFLD), age (25–64 years and ≥65 years), sex (male and female), race and ethnicity (Hispanic, non-Hispanic [NH]-White, NH-Black, NH-Asian including Pacific Islander, and NH-American Indian [AI]/Alaska Native [AN]) for analysis.

We employed the Joinpoint regression program to explore the trend in mortality rates among individuals with HCC, in order to analyze the annual percentage change (APC) and 95% confidence intervals (CIs) of age-standardized rates. The positivity/negativity and magnitude of APCs signify the direction and degree of trend changes. AutoARIMA is a widely used traditional time series prediction statistical model,[1, 2] and Prophet is an open source time series prediction statistical model developed by the Facebook team[3]. Both models are suitable for large-scale time series data prediction and analysis.

Auto Regressive Integrated Moving Average (ARIMA) technique is one of the commonly used approaches for time series investigation. The model captures the linear dependence and random volatility of the time series by combining three parts: autoregression (AR), difference (I) and moving average (MA). The AR section of the ARIMA model indicates that the current observation can be explained by a linear combination of its past values, represented as AR(p):

$$Y_t = \delta + \varphi_1 Y_{t-1} + \varphi_2 Y_{t-2} + \cdots + \varphi_p Y_{t-p} + \varepsilon_t$$

here, Y_t represents the value of the time series at time t, p is the order of the autoregressive term, $\varphi_1, \varphi_2, \cdots, \varphi_p$, are the autoregressive coefficients of the model, δ is the constant term, and ε_t is the error term at time t. The MA section of the ARIMA model indicates that the current observation is not only affected by the previous autoregressive part, but also by the linear combination of past random error terms, denoted as MA(q):

© 2024 Qiu S et al. *JAMA Network Open.*

$$Y_t = \mu + \varepsilon_t + \theta_1\varepsilon_{t-1} + \theta_2\varepsilon_{t-2} + \cdots + \theta_q\varepsilon_{t-q}$$

here, μ is the expected value of the time series, $\theta_1, \theta_2, \cdots, \theta_q$ are the sliding average coefficients, and q is the order of the sliding average term. Difference is used to deal with the non-stationarity of time series. Non-stationarity is usually manifested as the mean and variance of the time series changing over time. By performing a difference operation on the time series, the non-stationarity can be eliminated and a stationary series can be obtained. The mathematical expression of the difference operation is:

$$\Delta Y_t = Y_t - Y_{t-1} = Y_t - LY_t$$

So the expression of the ARIMA (p, d, q) model is:

$$(1 - \varphi_1 L - \varphi_1 L^2 - \cdots - \varphi_p L^p)\Delta^d Y_t = \delta + \varepsilon_t + \theta_1\varepsilon_{t-1} + \theta_2\varepsilon_{t-2} + \cdots + \theta_q\varepsilon_{t-q}$$

The AutoARIMA model is an automated extension of the ARIMA model that simplifies the model selection process. When using the R package for AutoARIMA modeling, the model order selection is usually based on a unit root test to determine the number of differences (d), and combined with information criteria (such as AIC) to optimize the model's autoregressive (p) and moving average (q) orders, using a step-wise procedure to traverse the model space, by gradually increasing or decreasing model parameters, to find the optimal model, thereby achieving a balance between accuracy and complexity. This approach greatly simplifies the model selection process while ensuring that the selected model can fit the data well.

During the modeling process, Prophet model decomposes time series data into multiple modules such as trend items, seasonal items, and holiday effects based on an additive model. After fitting each module, the predicted values of each module are combined based on the generalized additive model to obtain the prediction results of future time series, and provide a confidence interval for each predicted value to evaluate the reliability of the prediction results. Its algorithm model can be simply expressed as:

$$y(t) = g(t) + s(t) + h(t) + \varepsilon_t$$

here, $g(t)$ represents the trend term of the time series, $s(t)$ represents the seasonal term, $h(t)$ represents the holiday term, and ε_t represents the error term, which is usually assumed to follow a normal distribution. This is an additive form based on the decomposition method. Another form is a multiplicative form based on the decomposition method, which can be expressed as

$$y(t) = g(t) \times s(t) \times h(t) \times \varepsilon_t$$

Simply take the logarithm of the variables on both sides to convert the multiplicative form to the additive form.

We compared the root mean squared error (RMSE) of the two models to select the appropriate forecasting model. For HCV data, due to the shorter duration and fewer data

© 2024 Qiu S et al. *JAMA Network Open.*

points (starting from 2015), after selecting the forecasting model based on Root Mean Squared Error(RMSE) values, the forecast end year was chosen before the first negative value. If the AutoARIMA model is selected for prediction, we will perform a Ljung-Box test on its residuals. If the p-value of the Ljung-Box test is greater than 0.05, it means that the model fits the data well, that is, the residuals of the model appear as white noise, indicating that the model effectively captures the main structure of the data. RMSE is defined as:

$$RMSE = \sqrt{\frac{1}{n}\sum_{i=1}^{n}(\hat{y}_i - y_i)^2}$$

The National Cancer Institute’s Joinpoint Trend Analysis software version 5.0.2 and R 4.0.2 statistical software were used in this study. The significance threshold for two-sided p-values was set at 0.05.

eReferences

1.Box GEP. and Jenkins GM. (1970) Time series analysis: Forecasting and control. Holden-Day, San Francisco.

2.Hyndman R J, Khandakar Y. Automatic time series forecasting: the forecast package for R.Journal of statistical software, 2008, 27: 1-22.

3.Taylor SJ, Letham B. Forecasting at scale. The American Statistician, 2018, 72(1): 37-45.

© 2024 Qiu S et al. *JAMA Network Open.*

eTable 1. *International Statistical Classification of Diseases, Tenth Revision* (*ICD-10*), Codes Used in the Study

Disease	ICD-10
**Liver Cancer**	C22.0
**ALD**	K70.0, K70.1, K70.2, K70.3, K70.4, K70.9
**MASLD**	K75.8, K76.0
**HCV**	B17.1, B18.2
**HBV**	B16.0, B16.1, B16.2, B16.9, B17.0, B18.0, B18.1

Note: ALD, alcohol-associated liver disease; MASLD, metabolic dysfunction-associated steatotic liver disease; HCV, hepatitis C virus; HBV, hepatitis B virus.

© 2024 Qiu S et al. *JAMA Network Open.*

eTable 2. Root Mean Square Error (RMSE) Comparison and Model Selection Between AutoARIMA and Prophet Across Different Groups

Group		RMSE		Choice
		AutoARIMA	Prophet	
Overall				
	Overall	0.09	0.07	Prophet
	HCV	0.02	0.01	Prophet
	ALD	0.03	0.02	Prophet
	HBV	0.02	0.02	Prophet
	MASLD	0.01	0.01	AutoARIMA**
Sex				
Female	Overall	0.06	0.04	Prophet
	HCV	0.02	0.01	Prophet
	ALD	0.01	0.01	Prophet
	HBV	0.01	0.01	Prophet
	MASLD	0.01	0.01	Prophet
Male	Overall	0.14	0.13	Prophet
	HCV	0.03	0.00	Prophet
	ALD	0.06	0.04	Prophet
	HBV	0.02	0.02	Prophet
	MASLD	0.01	0.01	AutoARIMA**
Age				
25-64 years	Overall*	0.08	0.23	Prophet
	HCV	0.03	0.02	Prophet
	ALD	0.04	0.03	Prophet
	HBV	0.02	0.02	Prophet
	MASLD	0.00	0.00	Prophet
	Overall	0.35	0.17	Prophet

© 2024 Qiu S et al. *JAMA Network Open.*

≥65 years	HCV	0.07	0.03	Prophet
	ALD	0.05	0.03	Prophet
	HBV	0.02	0.02	Prophet
	MASLD	0.04	0.04	AutoARIMA**
Ethnicity				
Hispanics	Overall	0.42	0.29	Prophet
	HCV	0.14	0.08	Prophet
	ALD	0.06	0.05	Prophet
	MASLD	0.04	0.02	Prophet
NH-AI/AN	Overall	0.67	0.58	Prophet
	HCV	0.39	0.38	Prophet
	ALD	0.27	0.18	Prophet
NH-Asians	Overall	0.35	0.27	Prophet
	HCV	0.14	0.07	Prophet
	ALD	0.04	0.02	Prophet
	HBV	0.19	0.18	Prophet
NH-Blacks	Overall	0.30	0.24	Prophet
	HCV	0.16	0.09	Prophet
	ALD	0.06	0.04	Prophet
	HBV	0.02	0.02	Prophet
NH-Whites	Overall	0.05	0.05	Prophet
	HCV	0.05	0.00	Prophet
	ALD	0.02	0.02	Prophet
	HBV	0.01	0.01	AutoARIMA**
	MASLD	0.01	0.01	AutoARIMA**

*: Considering the 95% confidence interval, the overall trend, and the fact that the RMSE is not much different, this group uses Prophet with a larger RMSE.

© 2024 Qiu S et al. *JAMA Network Open.*

**: The AutoARIMA model was selected for prediction, and the p values of the Ljung-Box residual test were all greater than 0.05, indicating that the model fit well and the residuals had no significant autocorrelation.

© 2024 Qiu S et al. *JAMA Network Open.*

eTable 3. Characteristics of Hepatocellular Carcinoma (HCC)–Related Deaths in the US, 2006-2022

	Deaths					
**Characteristic**	**2006 to 2022**	**2006**	**2010**	**2015**	**2020**	**2022**
**Overall**	188,280 (100.0)	7,425 (100.0)	9,262 (100.0)	11,264 (100.0)	13,907 (100.0)	14,310 (100.0)
**Sex**						
Female	42,500 (22.6)	1,765 (23.8)	2,022 (21.8)	2,439 (21.6)	3,337 (24.0)	3,510 (24.5)
Male	145,780 (77.4)	5,660 (76.2)	7,240 (78.2)	8,825 (78.4)	10,570 (76.0)	10,800 (75.5)
**Age in years**						
25-64	83,488 (44.4)	3641 (49.0)	4838 (52.2)	5533 (49.1)	4697 (33.8)	4157 (29.1)
⩾65	104,792 (55.6)	3,784 (51.0)	4,424 (47.8)	5,731 (50.9)	9,210 (66.2)	10,153 (70.9)
**Etiology**						
HCV	42,111 (65.6)	1,269 (74.2)	2,211 (69.7)	3,052 (69.5)	2,615 (57.4)	2,319 (54.0)
ALD	14,176 (22.0)	201 (11.7)	578 (18.2)	876 (20.0)	1,270 (27.9)	1,281 (29.8)
HBV	5,369 (8.4)	228 (13.3)	338 (10.7)	329 (7.5)	315 (6.9)	317 (7.4)
MASLD	2,550 (4.0)	13 (0.8)	43 (1.4)	133 (3.0)	358 (7.8)	379 (8.8)

Data are *n* (%). Note: ALD, alcohol-associated liver disease; MASLD, metabolic dysfunction-associated steatotic liver disease; HCV, hepatitis C virus; HBV, hepatitis B virus.

© 2024 Qiu S et al. *JAMA Network Open.*

eTable 4. Observed and Projected Age-Standardized Mortality Rates (per 100 000) of Adults (Aged ≥25 years) With Hepatocellular Carcinoma (HCC), US, 2006-2040, Total and by Sex, Age, and Race and Ethnicity, and Further Stratified by HCC Etiology

Study Period		Etiology				
		Overall	***HCV****	***ALD***	***HBV***	***MASLD***
Overall						
**Observed**	2006	3.65	0.61	0.09	0.12	0.01
	2007	3.65	0.77	0.18	0.13	0.01
	2008	3.88	0.85	0.20	0.11	0.02
	2009	4.06	0.91	0.21	0.15	0.01
	2010	4.12	0.95	0.23	0.15	0.02
	2011	4.26	0.99	0.26	0.14	0.02
	2012	4.27	1.10	0.30	0.14	0.03
	2013	4.31	1.11	0.25	0.17	0.04
	2014	4.32	1.07	0.30	0.16	0.03
	2015	4.39	1.12	0.33	0.13	0.05
	2016	4.70	1.11	0.36	0.14	0.06
	2017	4.77	1.06	0.40	0.15	0.07
	2018	4.86	0.97	0.41	0.12	0.08
	2019	4.80	0.93	0.42	0.12	0.09
	2020	4.91	0.87	0.44	0.10	0.11
	2021	4.94	0.79	0.43	0.12	0.10
	2022	5.03	0.76	0.43	0.10	0.14
**Projected**	2023	5.11	0.69	0.50	0.12	0.13
	2024	5.22	0.64	0.53	0.11	0.15
	2025	5.31	0.57	0.52	0.13	0.15
	2026	5.33	0.51	0.55	0.12	0.16
	2027	5.40	0.45	0.58	0.11	0.17

© 2024 Qiu S et al. *JAMA Network Open.*

	2028	5.52	0.40	0.61	0.10	0.17
	2029	5.60	0.33	0.60	0.12	0.18
	2030	5.62	0.27	0.63	0.11	0.19
	2031	5.69	0.21	0.66	0.10	0.20
	2032	5.81	0.17	0.69	0.09	0.20
	2033	5.89	0.10	0.68	0.12	0.21
	2034	5.92	0.03	0.71	0.11	0.22
	2035	5.99	-	0.74	0.10	0.23
	2036	6.10	-	0.78	0.09	0.23
	2037	6.18	-	0.77	0.11	0.24
	2038	6.21	-	0.79	0.10	0.25
	2039	6.28	-	0.82	0.09	0.26
	2040	6.39	-	0.86	0.08	0.26
Sex						
Female						
**Observed**	2006	1.59	0.23	0.01	0.02	NA
	2007	1.53	0.24	0.05	0.04	0.01
	2008	1.58	0.25	0.04	0.04	0.02
	2009	1.67	0.31	0.05	0.02	0.01
	2010	1.66	0.33	0.04	0.03	0.02
	2011	1.73	0.35	0.05	0.05	0.02
	2012	1.72	0.37	0.04	0.05	0.03
	2013	1.80	0.40	0.04	0.05	0.02
	2014	1.77	0.36	0.05	0.04	0.03
	2015	1.79	0.36	0.06	0.04	0.04
	2016	2.00	0.36	0.07	0.04	0.04
	2017	1.95	0.33	0.08	0.04	0.04

© 2024 Qiu S et al. *JAMA Network Open.*

	2018	2.00	0.29	0.09	0.02	0.06
	2019	2.01	0.30	0.09	0.03	0.07
	2020	2.18	0.32	0.08	0.04	0.09
	2021	2.19	0.30	0.11	0.04	0.08
	2022	2.33	0.28	0.09	0.04	0.11
**Projected**	2023	2.31	0.27	0.10	0.04	0.10
	2024	2.39	0.28	0.11	0.05	0.11
	2025	2.45	0.25	0.11	0.04	0.10
	2026	2.49	0.24	0.12	0.04	0.12
	2027	2.54	0.24	0.12	0.04	0.13
	2028	2.62	0.24	0.12	0.05	0.14
	2029	2.68	0.21	0.13	0.04	0.13
	2030	2.72	0.20	0.14	0.04	0.14
	2031	2.78	0.20	0.14	0.05	0.15
	2032	2.86	0.20	0.14	0.05	0.16
	2033	2.92	0.17	0.15	0.04	0.16
	2034	2.96	0.16	0.16	0.04	0.17
	2035	3.02	0.16	0.16	0.05	0.18
	2036	3.09	0.16	0.16	0.05	0.19
	2037	3.15	0.13	0.17	0.04	0.18
	2038	3.19	0.12	0.17	0.05	0.19
	2039	3.25	0.12	0.18	0.05	0.20
	2040	3.33	0.12	0.18	0.05	0.21
Male						
**Observed**	2006	6.10	1.00	0.16	0.20	0.01
	2007	6.18	1.30	0.38	0.26	0.02
	2008	6.57	1.45	0.38	0.23	0.02

© 2024 Qiu S et al. *JAMA Network Open.*

	2009	6.82	1.55	0.37	0.25	0.02
	2010	7.01	1.59	0.46	0.27	0.02
	2011	7.16	1.69	0.49	0.24	0.03
	2012	7.15	1.86	0.54	0.24	0.05
	2013	7.18	1.87	0.51	0.25	0.05
	2014	7.20	1.90	0.62	0.25	0.05
	2015	7.37	1.96	0.62	0.25	0.06
	2016	7.85	1.92	0.73	0.21	0.08
	2017	8.01	1.86	0.76	0.23	0.10
	2018	8.08	1.75	0.78	0.21	0.11
	2019	8.02	1.61	0.80	0.24	0.11
	2020	8.06	1.47	0.83	0.22	0.15
	2021	8.10	1.36	0.80	0.20	0.18
	2022	8.15	1.28	0.82	0.21	0.16
**Projected**	2023	8.30	1.18	0.95	0.22	0.17
	2024	8.45	1.07	1.01	0.21	0.18
	2025	8.53	0.96	0.99	0.21	0.19
	2026	8.55	0.88	1.05	0.21	0.19
	2027	8.64	0.79	1.11	0.21	0.20
	2028	8.78	0.68	1.17	0.20	0.21
	2029	8.86	0.57	1.15	0.21	0.22
	2030	8.88	0.49	1.20	0.20	0.23
	2031	8.97	0.39	1.26	0.20	0.24
	2032	9.11	0.28	1.32	0.20	0.25
	2033	9.20	0.17	1.30	0.20	0.26
	2034	9.22	0.09	1.36	0.20	0.27
	2035	9.30	-	1.42	0.19	0.28

© 2024 Qiu S et al. *JAMA Network Open.*

	2036	9.45	-	1.48	0.19	0.29
	2037	9.53	-	1.46	0.19	0.30
	2038	9.55	-	1.51	0.19	0.31
	2039	9.63	-	1.57	0.18	0.31
	2040	9.78	-	1.63	0.18	0.32
Age						
25-64 years						
**Observed**	2006	2.06	0.55	0.06	0.09	NA
	2007	2.12	0.71	0.16	0.11	NA
	2008	2.28	0.78	0.17	0.08	NA
	2009	2.45	0.83	0.18	0.12	NA
	2010	2.43	0.88	0.23	0.12	0.01
	2011	2.60	0.92	0.21	0.12	0.01
	2012	2.61	0.99	0.25	0.12	0.01
	2013	2.60	0.99	0.20	0.15	0.01
	2014	2.53	0.92	0.25	0.13	0.01
	2015	2.51	0.91	0.26	0.10	0.02
	2016	2.54	0.90	0.28	0.10	0.02
	2017	2.46	0.79	0.30	0.12	0.02
	2018	2.36	0.68	0.30	0.08	0.02
	2019	2.16	0.61	0.31	0.08	0.03
	2020	2.07	0.51	0.28	0.08	0.03
	2021	1.95	0.42	0.27	0.08	0.03
	2022	1.79	0.37	0.26	0.08	0.03
**Projected**	2023	2.17	0.27	0.34	0.08	0.04
	2024	2.18	0.20	0.36	0.07	0.04
	2025	2.13	0.10	0.34	0.10	0.04

© 2024 Qiu S et al. *JAMA Network Open.*

	2026	2.11	0.01	0.36	0.09	0.04
	2027	2.10	-	0.38	0.08	0.05
	2028	2.11	-	0.40	0.07	0.05
	2029	2.06	-	0.38	0.09	0.05
	2030	2.04	-	0.40	0.08	0.05
	2031	2.03	-	0.42	0.07	0.05
	2032	2.04	-	0.44	0.06	0.06
	2033	1.99	-	0.43	0.08	0.06
	2034	1.97	-	0.45	0.07	0.06
	2035	1.96	-	0.47	0.06	0.06
	2036	1.97	-	0.48	0.05	0.07
	2037	1.92	-	0.47	0.08	0.07
	2038	1.90	-	0.49	0.06	0.07
	2039	1.89	-	0.51	0.06	0.07
	2040	1.90	-	0.53	0.05	0.08
≥65 years						
**Observed**	2006	10.20	0.86	0.23	0.22	NA
	2007	9.95	0.99	0.28	0.26	0.03
	2008	10.44	1.15	0.38	0.23	0.07
	2009	10.71	1.24	0.32	0.25	0.05
	2010	11.08	1.22	0.30	0.29	0.07
	2011	11.08	1.28	0.44	0.24	0.08
	2012	11.08	1.59	0.49	0.26	0.10
	2013	11.39	1.62	0.46	0.23	0.14
	2014	11.71	1.69	0.52	0.28	0.15
	2015	12.15	1.98	0.62	0.25	0.19
	2016	13.58	1.98	0.70	0.26	0.26

© 2024 Qiu S et al. *JAMA Network Open.*

	2017	14.28	2.15	0.81	0.26	0.29
	2018	15.14	2.16	0.86	0.23	0.36
	2019	15.68	2.28	0.93	0.30	0.41
	2020	16.59	2.32	1.06	0.28	0.50
	2021	17.25	2.32	1.10	0.25	0.46
	2022	18.37	2.34	1.14	0.28	0.56
**Projected**	2023	19.02	2.39	1.24	0.28	0.59
	2024	19.81	2.42	1.35	0.28	0.63
	2025	20.71	2.47	1.39	0.27	0.66
	2026	21.49	2.49	1.46	0.28	0.70
	2027	22.27	2.52	1.55	0.29	0.73
	2028	23.06	2.55	1.66	0.29	0.77
	2029	23.96	2.60	1.69	0.28	0.80
	2030	24.74	2.63	1.76	0.29	0.84
	2031	25.52	2.66	1.85	0.30	0.87
	2032	26.31	2.69	1.97	0.30	0.91
	2033	27.21	2.73	2.00	0.29	0.94
	2034	27.99	2.76	2.07	0.30	0.98
	2035	28.77	2.79	2.16	0.31	1.01
	2036	29.56	2.82	2.27	0.31	1.05
	2037	30.46	2.87	2.30	0.30	1.08
	2038	31.24	2.90	2.37	0.31	1.12
	2039	32.02	2.93	2.47	0.32	1.15
	2040	32.81	2.95	2.58	0.32	1.19
Ethnicity						
Hispanics						
**Observed**	2006	6.49	1.08	0.25	NA	NA

© 2024 Qiu S et al. *JAMA Network Open.*

	2007	5.89	1.29	0.40	NA	NA
	2008	6.35	1.42	0.45	NA	NA
	2009	6.61	1.42	0.52	NA	NA
	2010	6.44	1.47	0.46	NA	NA
	2011	6.87	1.61	0.55	NA	NA
	2012	6.88	1.79	0.64	NA	0.08
	2013	6.78	1.73	0.61	NA	0.07
	2014	6.35	1.61	0.71	NA	0.07
	2015	6.39	1.56	0.75	NA	0.14
	2016	7.32	1.65	0.81	NA	0.09
	2017	7.25	1.50	0.89	NA	0.12
	2018	7.68	1.51	0.81	NA	0.17
	2019	6.99	1.25	0.87	NA	0.17
	2020	7.18	1.09	0.83	NA	0.16
**Projected**	2021	7.49	1.14	0.98	NA	0.18
	2022	7.44	1.01	1.01	NA	0.20
	2023	7.48	0.89	1.05	NA	0.22
	2024	7.62	0.76	1.10	NA	0.23
	2025	7.80	0.75	1.15	NA	0.24
	2026	7.75	0.62	1.18	NA	0.25
	2027	7.79	0.50	1.22	NA	0.27
	2028	7.93	0.37	1.27	NA	0.28
	2029	8.11	0.36	1.32	NA	0.29
	2030	8.06	0.23	1.35	NA	0.31
	2031	8.10	0.11	1.39	NA	0.32
	2032	8.24	-	1.43	NA	0.33
	2033	8.42	-	1.48	NA	0.34

© 2024 Qiu S et al. *JAMA Network Open.*

	2034	8.37	-	1.51	NA	0.36
	2035	8.41	-	1.55	NA	0.37
	2036	8.55	-	1.60	NA	0.39
	2037	8.73	-	1.65	NA	0.39
	2038	8.68	-	1.68	NA	0.41
	2039	8.72	-	1.72	NA	0.43
	2040	8.86	-	1.77	NA	0.44
NH-AI/AN						
**Observed**	2006	5.46	NA	NA	NA	NA
	2007	6.49	1.01	NA	NA	NA
	2008	5.81	1.79	NA	NA	NA
	2009	5.31	1.05	NA	NA	NA
	2010	7.47	1.64	0.81	NA	NA
	2011	6.01	1.51	0.65	NA	NA
	2012	8.13	1.78	0.75	NA	NA
	2013	7.80	2.12	0.58	NA	NA
	2014	6.90	1.81	1.27	NA	NA
	2015	7.92	2.34	0.84	NA	NA
	2016	8.60	1.81	1.38	NA	NA
	2017	8.39	1.93	0.91	NA	NA
	2018	8.57	1.95	1.30	NA	NA
	2019	8.37	1.26	1.58	NA	NA
	2020	9.88	2.47	1.69	NA	NA
**Projected**	2021	9.21	1.70	1.45	NA	NA
	2022	9.60	1.73	1.65	NA	NA
	2023	10.05	1.77	1.84	NA	NA
	2024	10.56	1.80	2.01	NA	NA

	2025	10.25	1.55	1.82	NA	NA
	2026	10.63	1.58	2.02	NA	NA
	2027	11.08	1.62	2.20	NA	NA
	2028	11.60	1.65	2.38	NA	NA
	2029	11.29	1.40	2.18	NA	NA
	2030	11.67	1.43	2.38	NA	NA
	2031	12.12	1.47	2.57	NA	NA
	2032	12.63	1.50	2.74	NA	NA
	2033	12.32	1.25	2.55	NA	NA
	2034	12.71	1.28	2.74	NA	NA
	2035	13.16	1.32	2.93	NA	NA
	2036	13.67	1.35	3.10	NA	NA
	2037	13.36	1.10	2.91	NA	NA
	2038	13.74	1.13	3.11	NA	NA
	2039	14.19	1.16	3.29	NA	NA
	2040	14.71	1.20	3.47	NA	NA
NH-Asians						
**Observed**	2006	8.38	1.05	NA	1.26	NA
	2007	7.65	0.99	NA	1.46	NA
	2008	8.38	1.44	NA	1.32	NA
	2009	8.33	1.37	NA	1.51	NA
	2010	8.34	1.26	0.13	1.91	NA
	2011	7.80	1.18	0.15	1.55	NA
	2012	7.58	1.36	0.16	1.60	NA
	2013	7.19	1.08	0.11	1.50	NA
	2014	6.84	0.99	0.14	1.46	NA
	2015	6.78	1.09	0.09	1.22	NA

© 2024 Qiu S et al. *JAMA Network Open.*

	2016	6.84	0.80	0.19	1.30	NA
	2017	6.64	0.84	0.14	1.28	NA
	2018	6.50	0.78	0.18	0.99	NA
	2019	6.33	0.80	0.21	1.05	NA
	2020	6.45	0.61	0.22	1.08	NA
**Projected**	2021	6.09	0.61	0.17	1.16	NA
	2022	5.83	0.53	0.19	1.10	NA
	2023	5.67	0.45	0.22	1.04	NA
	2024	5.62	0.34	0.24	0.99	NA
	2025	5.44	0.33	0.20	1.04	NA
	2026	5.18	0.26	0.22	0.98	NA
	2027	5.02	0.17	0.24	0.92	NA
	2028	4.97	0.07	0.27	0.87	NA
	2029	4.79	0.06	0.22	0.92	NA
	2030	4.53	-	0.25	0.85	NA
	2031	4.38	-	0.27	0.80	NA
	2032	4.33	-	0.29	0.74	NA
	2033	4.15	-	0.25	0.79	NA
	2034	3.89	-	0.27	0.73	NA
	2035	3.73	-	0.30	0.67	NA
	2036	3.68	-	0.32	0.62	NA
	2037	3.50	-	0.28	0.67	NA
	2038	3.24	-	0.30	0.60	NA
	2039	3.08	-	0.32	0.55	NA
	2040	3.03	-	0.34	0.49	NA
NH-Blacks						
**Observed**	2006	5.44	1.28	0.07	0.20	NA

	2007	5.36	1.50	0.18	0.22	NA
	2008	5.73	1.63	0.22	0.24	NA
	2009	6.32	1.90	0.30	0.26	NA
	2010	6.22	1.96	0.28	0.25	NA
	2011	6.37	2.08	0.28	0.21	NA
	2012	6.37	2.10	0.29	0.24	NA
	2013	6.51	2.23	0.31	0.27	NA
	2014	6.28	2.14	0.34	0.23	NA
	2015	6.24	2.11	0.26	0.23	NA
	2016	6.99	2.31	0.39	0.18	NA
	2017	6.76	2.12	0.45	0.22	NA
	2018	6.85	1.94	0.45	0.24	NA
	2019	6.68	1.74	0.42	0.23	NA
	2020	6.41	1.67	0.39	0.22	NA
**Projected**	2021	7.18	1.61	0.51	0.24	NA
	2022	7.09	1.44	0.51	0.23	NA
	2023	7.09	1.32	0.51	0.22	NA
	2024	7.19	1.23	0.52	0.21	NA
	2025	7.53	1.14	0.59	0.24	NA
	2026	7.43	0.98	0.59	0.23	NA
	2027	7.44	0.85	0.60	0.22	NA
	2028	7.54	0.76	0.61	0.21	NA
	2029	7.87	0.67	0.67	0.24	NA
	2030	7.78	0.51	0.68	0.23	NA
	2031	7.79	0.38	0.68	0.22	NA
	2032	7.89	0.30	0.69	0.21	NA
	2033	8.22	0.20	0.76	0.24	NA

© 2024 Qiu S et al. *JAMA Network Open.*

	2034	8.13	0.04	0.76	0.23	NA
	2035	8.13	-	0.77	0.22	NA
	2036	8.24	-	0.78	0.21	NA
	2037	8.57	-	0.84	0.24	NA
	2038	8.47	-	0.85	0.22	NA
	2039	8.48	-	0.85	0.21	NA
	2040	8.58	-	0.86	0.21	NA
NH-Whites						
**Observed**	2006	2.91	0.44	0.08	0.05	0.01
	2007	3.02	0.57	0.15	0.05	0.01
	2008	3.15	0.63	0.19	0.05	0.02
	2009	3.24	0.68	0.17	0.06	0.02
	2010	3.36	0.69	0.22	0.05	0.02
	2011	3.47	0.74	0.21	0.06	0.02
	2012	3.44	0.83	0.24	0.03	0.03
	2013	3.53	0.86	0.21	0.06	0.04
	2014	3.59	0.88	0.27	0.03	0.03
	2015	3.70	0.92	0.28	0.05	0.05
	2016	3.89	0.85	0.32	0.03	0.07
	2017	4.01	0.86	0.32	0.03	0.07
	2018	4.00	0.77	0.36	0.03	0.08
	2019	4.08	0.76	0.36	0.04	0.09
	2020	4.19	0.72	0.38	0.03	0.12
**Projected**	2021	4.30	0.77	0.39	0.04	0.13
	2022	4.36	0.74	0.41	0.03	0.13
	2023	4.45	0.71	0.44	0.04	0.14
	2024	4.56	0.68	0.46	0.03	0.15

© 2024 Qiu S et al. *JAMA Network Open.*

	2025	4.65	0.73	0.46	0.04	0.16
	2026	4.72	0.70	0.49	0.03	0.17
	2027	4.80	0.67	0.51	0.04	0.17
	2028	4.91	0.64	0.54	0.03	0.18
	2029	5.01	0.69	0.53	0.04	0.19
	2030	5.07	0.66	0.56	0.03	0.20
	2031	5.16	0.63	0.59	0.04	0.20
	2032	5.27	0.60	0.61	0.03	0.21
	2033	5.36	0.65	0.61	0.04	0.22
	2034	5.42	0.62	0.63	0.03	0.23
	2035	5.51	0.59	0.66	0.04	0.24
	2036	5.62	0.56	0.69	0.04	0.24
	2037	5.71	0.61	0.68	0.04	0.25
	2038	5.78	0.58	0.71	0.04	0.26
	2039	5.87	0.55	0.73	0.04	0.27
	2040	5.97	0.52	0.76	0.04	0.28

*:The projections of overall, sex and age group are based on data from 2015 to 2022, the projections by race and ethnicity are based on data from 2015 to 2020.

Note: ALD, alcohol-associated liver disease; MASLD, metabolic dysfunction-associated steatotic liver disease; HCV, hepatitis C virus; HBV, hepatitis B virus

© 2024 Qiu S et al. *JAMA Network Open.*

eFigure. Causes of Deaths Related to Hepatocellular Carcinoma (HCC) Presented by the Age-Standardized Mortality Rate (ASMR) per 100 000 and Predicted Value to 2040, Overall and by Race and Ethnicity

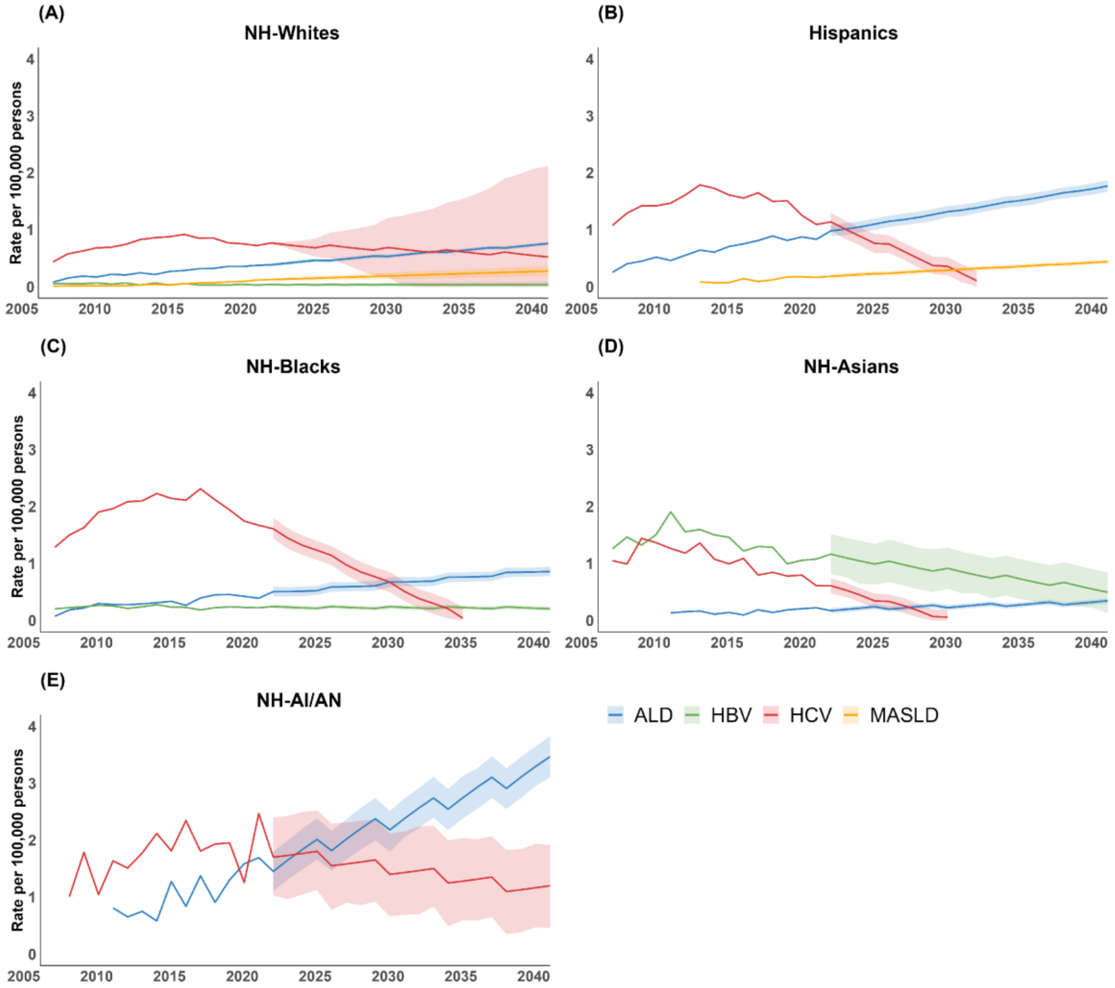

Note: The hepatitis C virus (HCV) group is projected to 2040 based on data from 2015 to 2020. The other groups are projected to 2040 based on data from 2006 to 2020. ALD, alcohol-associated liver disease; MASLD, metabolic dysfunction-associated steatotic liver disease; HCV, hepatitis C virus; HBV, hepatitis B virus

© 2024 Qiu S et al. *JAMA Network Open.*
