# Supplementary material for: Trends in Hepatocellular Carcinoma Mortality Rates in the US and Projections Through 2040
Source: JAMA Netw Open. 2024 Nov 18;7(11):e2445525. doi: 10.1001/jamanetworkopen.2024.45525 (PMC11574689; doi:10.1001/jamanetworkopen.2024.45525)
Supplement: Supplement 2. — Data Sharing Statement [file jamanetwopen-e2445525-s002.pdf]

Data Sharing Statement

Qiu. Trends in Hepatocellular Carcinoma Mortality Rates in the US and Projections Through 2040. *JAMA Netw Open*. Published November 18, 2024.
doi:10.1001/jamanetworkopen.2024.45525

Data
Data available: The NVSS can be accessed through this website: https://wonder.cdc.gov/mcd.html.
